# Different long-term oncologic outcomes after radical surgical resection for neuroendocrine carcinoma and adenocarcinoma of the stomach

**DOI:** 10.18632/oncotarget.15488

**Published:** 2017-02-18

**Authors:** Jian-Wei Xie, Jun Lu, Jian-Xian Lin, Chao-Hui Zheng, Ping Li, Jia-Bin Wang, Qi-Yue Chen, Long-Long Cao, Mi Lin, Ru-Hong Tu, Chang-Ming Huang

**Affiliations:** ^1^ Department of Gastric Surgery, Fujian Medical University Union Hospital, Fuzhou, Fujian, People's Republic of China

**Keywords:** gastric neoplasm, neuroendocrine neoplasm, prognosis, propensity score matching study

## Abstract

**Purpose:**

To explore differences in long-term outcomes between gastric neuroendocrine carcinoma (GNEC) and gastric adenocarcinoma (GAC).

**Methods:**

One hundred GNEC patients and 3089 GAC patients were enrolled. Differences in long-term outcomes between the groups were analyzed by 1:2 propensity score matching.

**Results:**

Statistically significant differences between the groups were noted in terms of gender, American Society of Anesthesiologists score, tumor size, T stage, N stage, TNM stage and surgical approach. However, differences were not significant after matching. The 3-year and 5-year overall survival rates for the GNEC group were reduced compared with those for the GAC group, though disease-free survival rates and mean recurrence times were similar. Notably, the mean post-recurrence survival of the GNEC group was significantly worse than that of the GAC group (5.2 *vs*. 14.8 months, *p*<0.001). A strong negative correlation was found between a high Ki-67 labeling index and overall survival time. Cox regression analysis indicated the Ki-67 labeling index to be an independent factor influencing patient post-recurrence survival.

**Conclusions:**

The long-term oncologic outcome of GNEC was worse than that of GAC, which may be relative to its reduced post-recurrence survival. A high Ki-67 labeling index was an independent factor influencing patient post-recurrence survival.

## INTRODUCTION

Gastric neuroendocrine carcinoma (GNEC), which accounts for 0.16 to 1.48% of all gastric cancers [1–3], is derived from gastric epithelial tissues and has the characteristics of neuroendocrine differentiation tumors. Given its low incidence, relevant studies on GNEC are limited. GNEC differs significantly from gastric adenocarcinoma (GAC) based on biological characteristics and pathological features. GNEC is more likely to be accompanied by vessel invasion, lymph node metastasis and distant metastasis, which indicate a poor prognosis. For treating GNEC, National Comprehensive Cancer Network (NCCN; 2014) guidelines also recommend radical resection and cleaning of lymph nodes around the stomach. Only a few studies on GNEC long-term outcomes with radical surgery have been conducted to date, limiting further exploration and treatment options for this rare and highly malignant neoplasm. In this study, we analyzes differences in long-term outcomes after radical surgery between a GNEC group and a GAC group based on propensity score matching (PSM) and evaluated impact factors influencing the different outcomes.

## RESULTS

### Patient characteristics

The clinicopathological parameters of all patients (*n* = 3189) are outlined in Table 1. The proportions of male gender, tumor size, T stage, lymph node metastasis rate and total gastrectomy in the GNEC group were increased compared with the GAC group, whereas average American Society of Anesthesiologists (ASA) scores were reduced. In total, 97 GNEC and 194 GAC patients were selected by a 1:2 PSM. Differences in the clinicopathological parameters of post-matched samples were not significant. Of the 97 GNEC cases, 25 were of the small cell type and 72 of the large cell type. The clinicopathological factors significantly associated with the prognosis of the 97 GNEC patients are listed in Table 2.

**Table 1:** Patient characteristics and treatment details

	All Patients			Propensity-Matched Patients		
Variables	GAC (*n* = 3089)	GNEC (*n* = 100)	*p*	GAC (*n* = 194)	GNEC (*n* = 97)	*p*
Age (year)			0.114			0.736
<65	2057	59		114	59	
≥65	1032	41		80	38	
Gender			0.001			0.406
Male	2274	48		106	48	
Female	815	52		88	49	
ASA score			0.005			0.874
1	1901	46		84	45	
2	1015	44		94	44	
≥3	173	10		16	8	
Tumor size (cm)			0.007			0.392
<50	1503	35		76	33	
≥50	1586	65		118	64	
T stage			0.001			0.247
T1	681	1		4	1	
T2	351	12		38	12	
T3	761	80		134	78	
T4	1296	7		18	6	
N stage			0.008			0.332
N0	1075	22		50	20	
N1	2014	78		144	77	
Operation types			0.012			0.131
Total gastrectomy	1660	68		112	67	
Distal gastrectomy	1368	29		80	29	
Proximal gastrectomy	61	2		2	1	
Surgical method			0.052			0.610
Laparoscopic	2078	58		120	57	
Open	1011	42		74	40	

**Table 2:** Univariate and multivariate analyses of variables for 97 GNEC patients

		Univariate analysis			Multivariate analysis		
Variables	*N* (%)	3-yearsurvival rate (%)	5-year survival rate (%)	*P*	HR	95%CI	*P*
N	97 (100%)						
Age				0.727			
<65	59 (61%)	51.6	38.5				
≥65	38 (39%)	59.4	33.6				
Gender				0.499			
Male	48 (49%)	56.2	38.9				
Female	49 (51%)	53.3	37.1				
Location of tumor				0.071			
Upper	49 (50%)	44.7	31.9				
Middle	25 (26%)	62.1	44.1				
Lower	23 (24%)	71.5	52.1				
Classification				0.903			
Small cell	25 (26%)	61.1	46.0				
Large cell	72 (74%)	52.4	36.7				
Tumor diameter (cm)				0.017			0.438
<5	35 (36%)	72.0	60.2				
≥5	62 (64%)	45.3	27.5				
pT				0.09			
T1	1 (1%)	100	100				
T2	12 (12%)	82.5	78.6				
T3	78 (81%)	49.4	28.6				
T4	6 (6%)	50.0	50.0				
pN				0.005			
N0	20 (21%)	90.0	57.6		reference		
N1	77 (79%)	45.3	27.3		3.494	1.352-9.031	0.010
pTNM				0.018			0.852
I	1 (1%)	100	100				
II	19 (19%)	71.6	57.3				
III	77 (80%)	45.3	27.8				
Chemotherapy				0.000			
Yes	44 (45%)	74.2	58.9		0.381	0.203-0.714	0.003
No	53 (55%)	37.3	19.2		reference		
Surgical approach				0.298			
LG#	57 (59%)	46.9	35.7				
OG#	40 (41%)	64.5	44.3				
NEC components				0.700			
<70%	42 (43%)	56.2	39.9				
≥70%	55 (57%)	53.8	37.2				
Mitotic count (/10 HPF#)				0.017			0.151
<36	57 (59%)	60.4	46.1				
≥36	40 (41%)	44.8	22.4				
Ki-67 (%)				0.004			
<57.5%	33 (34%)	68.0	56.1		reference		
≥57.5%	64 (66%)	48.7	27.0		2.384	1.131-5.025	0.022

#: LG - laparoscopic gastrectomy, OG - open gastrectomy, NEC - gastric neuroendocrine carcinoma, HPF – high-power field

### Comparisons of overall and recurrence-free survival rates

**Figure 1:**
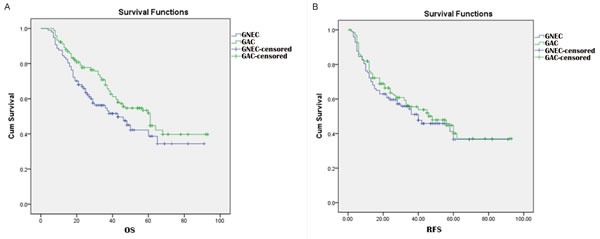
Comparison of OS **A.** and RFS **B.** curves for GNEC and GAC.

The median follow-up periods were 47 and 48 months for the GNEC and GAC groups. Compared with the GAC group, both the 3-year survival (54.7% *vs*. 67.9%, *p* = 0.018) 5-year survival (38.7% *vs*. 51.8%, *p* = 0.030) rates of the GNEC group were poorer (Figure 1A). However, similar 3-year recurrence-free survival (RFS) (51.1% *vs*. 55.5%, *p* = 0. 628) and 5-year RFS (36.6% *vs*. 40.1%, *p* = 0. 345) rates were observed for both groups (Figure 1B).

### Recurrence and treatment

**Figure 2:**
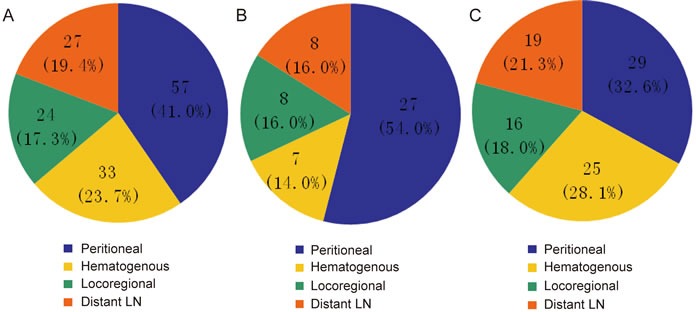
Recurrence types: **A.** overall recurrence types; **B.** recurrence types of GNEC; **C.** recurrence types of GAC.

There were 152 cases without recurrence and 139 cases with recurrence; the latter included 50 GNEC patients and 89 GAC patients. The recurrence rates of the two groups were similar (51.5% *vs*. 45.9%, *p* > 0.05). The mean recurrence time was also similar between the groups (16.5 months *vs*. 18.7 months, *p* = 0.810). The types of recurrence for GNEC and GAC are presented in Figure 2. In the GNEC group, peritoneal recurrence was the most common type, followed by distant nodal metastasis, local recurrence and hematogenous metastasis. Peritoneal recurrence was also the most common type in the GAC group, followed by hematogenous metastasis, distant nodal metastasis and local recurrence. In addition, 40% (20/50) of the GNEC patients with recurrence received chemotherapy, and 35% (35/99) of the GAC patients with recurrence did not. No significant difference in the proportion receiving chemotherapy was noted between the groups (*p* > 0.05).

### Analysis of postrecurrence survival (PRS)

**Figure 3:**
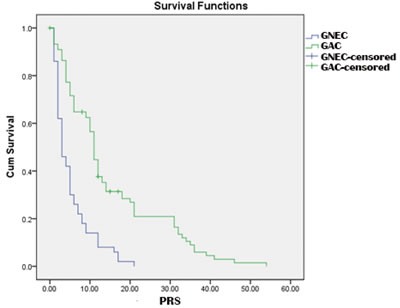
Comparison of PRS curves for GNEC and GAC

The overall mean PRS was 10.4 months. As shown in Figure 3, there were significant differences in PRS between the GNEC and GAC groups (5.2 *vs*. 14.8 months, *p* < 0.001), with the 1- and 2-year PRS rates of the GNEC group being reduced compared with the GAC group (14.0% *vs*. 37.7%, *p* < 0.001; 0% *vs*. 20.9%, *p* = 0.002), and an improved PRS time was noted in patients who received chemotherapy after recurrence (13.9 *vs*. 9.8 months, *p* = 0.044). However, according to stratified analysis, PRS in the GNEC group was similar between patients who had received chemotherapy and those who had not (6.4 *vs*. 4.4 months, *p* = 0.202). Conversely, significant differences were noted in the GAC group (18.3 *vs*. 12.8 months, *p* = 0.049).

### Prognostic value of the Ki-67 labeling index for GNEC

**Figure 4:**
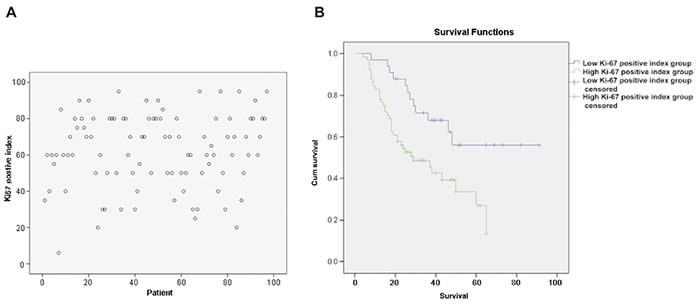
**A.** Distribution of the Ki-67 labeling index; **B.** relationship between the Ki-67 labeling index and GNEC patient survival time.

According to receiver operating characteristic (ROC) curve calculations, the sum of the sensitivity and specificity of the Ki-67 labeling index reached its maximum at a cut-off of 57.5%. The distribution of the Ki-67 labeling index is shown in Figure 4A. We divided GNEC patients into two groups: 33 cases with a low Ki-67 labeling index ( < 57.5%) and 64 cases with a high Ki-67 labeling index ( ≥ 57.5%). As shown in Figure 4B, the 5-year overall survival (OS) of the high Ki-67 labeling index group was significantly reduced compared with that of the low Ki-67 labeling index group (27.0% *vs*. 56.1%, *p* = 0.004), as was the 5-year RFS (61.2% *vs*. 22.7%, *p* = 0.014). In addition, the recurrence rate of the high Ki-67 labeling index group was significantly increased compared with that of the low Ki-67 labeling index group (59.3% *vs*. 36.4%, *p* = 0.032), whereas the PRS of the high Ki-67 labeling index group was significantly greater than that of the low Ki-67 labeling index group (8.9 months *vs*. 4.0 months, *p* = 0.004).

### Analysis of factors influencing the PRS of GNEC

As listed in Table 3, univariate and multivariate analyses indicated that the Ki-67 labeling index is an independent prognostic factor for GNEC patients with recurrence.

**Table 3:** Prognostic factors for the PRS of 139 recurrence patients

	Univariate analysis		Multivariate analysis		
Variables	Patients	Patients	OR (95%CI)	*P*-value
Ages (year)		0.236		
<65	86			
≥65	53			
Gender		0.710		
Mal	96			
Female	43			
ASA score		0.161		
1	58			
2	67			
≥3	14			
Tumor size (cm)		0.915		
<50	42			
≥50	97			
T stage		0.767		
T1	0			
T2	10			
T3	114			
T4	15			
N stage		0.274		
N0	17			
N1	122			
Operation types		0.332		
Totalgastrectomy	96			
Distal gastrectomy	40			
Proximal gastrectomy	3			
Surgical methods		0.626		
Laparoscopic	86			
Open	53			
Ki-67 labeling index (%)		0.000		0.000
<52.5	76		1 (reference)	
≥52.5	63			
Treatment after recurrence		0.044		.062
Chemotherapy	55		1 (reference)	
Best supportive care	84			
RFS		0.051		
<12 months	58			
≥12 months	81			

## DISCUSSION

The incidence of GNEC is increasing, and the prognosis is poor [4–6]. Because GNEC easily infiltrates vessels and lymphatic vessels, the OS of GNEC is poorer than that of GAC [7]. Jiang et al. [3] reported that the 5-year survival rate of 42 large cell-type GNEC patients (31.1%) was reduced compared with 307 GAC patients (69.3%). Furthermore, when comparing tumor stages, the survival rate of stage I-IV GNEC was significantly worse than that of GAC. The present study found a markedly worse 5-year survival rate for the GNEC group (38.7%) compared to the GAC group (51.8%). Although our study revealed a similar RFS between the groups, the OS of the GNEC group was significantly lower, which might be related to the reduced PRS of these patients. Treatment of recurrence is an important factor influencing PRS [8, 9], and recurrence patients receiving chemotherapy and/or radiotherapy exhibited increased PRS rates compared with those who underwent best support treatment [10]. Nonetheless, no criteria are available regarding the treatment of recurrent GNEC. Based on the treatment criteria of small cell lung cancer, Okita et al. used a treatment based on a combination of cisplatin and irinotecan in patients with recurrent GNEC after radical resection, and the overall response to chemotherapy was 75% [11]. We adopted chemotherapy based on fluorouracil (5-Fu) to treat recurrent GNEC patients and found that the PRS of these patients who received chemotherapy was similar to that of patients who had not received chemotherapy. Indeed, the results indicated a lack of improved PRS in recurrent GNEC patients who received 5-Fu chemotherapy. This finding prompted us not to follow the treatment experiences of GAC for GNEC. However, combination treatment based on cisplatin and irinotecan as well as capecitabine in combination with temozolomide was able to improve the outcome of recurrent GNEC patients [11, 12].

The Ki-67 labeling index is related to GNEC recurrence and prognosis [13–15]. Boo et al. [16] demonstrated that a high Ki-67 labeling index and poor differentiation are closely related to tumor recurrence. Multivariate analysis indicated the Ki-67 labeling index to be the only independent prognostic factor. Our study showed an increased recurrence rate for the high Ki-67 labeling index group ( ≥ 57.5%) with reduced OS time. The prognosis of GNEC also appears to depend on a different type of chemotherapy schedule, targeted on the basis of Ki-67 value (more or less than 55%) [17]. Moreover, the Ki-67 labeling index is related to the chemotherapy outcome of metastatic or recurrent GNEC [18]. By analyzing data from 252 advanced gastrointestinal neuroendocrine tumor patients who were given chemotherapy, Sorbye found that the 30-month survival rate was 23% when the patients had a Ki-67 labeling index < 55%, which was only 7% for those with a Ki-67 labeling index ≥ 55% [19]. Our study revealed a significantly reduced PRS for the high Ki-67 labeling index group compared with the low Ki-67 labeling index group and that the Ki-67 labeling index was an independent factor influencing the PRS of GNEC. As the results indicated that GNEC patients with a high Ki-67 labeling index have a higher propensity for recurrence, it is necessary to enhance follow-up surveillance and identify potential metastasis and recurrence as soon as possible.

To the best of our knowledge, this is the first study adopting PSM to compare the outcome of radical resection in GNEC and GAC patients. The offset is unavoidable, although we selected the PSM. Therefore, it is necessary to further evaluate the outcome of radical surgery for GNEC using high-quality, multi-center prospective trials.

## MATERIALS AND METHODS

### Patients

This study was a retrospective analysis of prospectively maintained clinical pathologic data derived from 100 GNEC patients and 3089 GAC patients at the Department of Gastric Surgery, Fujian Medical University Union Hospital between January 2006 and December 2013. Two groups were matched for gender, age, ASA score, operative period, tumor size and T and N stages. In total, 97 GNEC patients and 194 GAC patients were included using 1:2 PSM. GNEC staging was classified according to World Health Organization (WHO) gastroenteropancreatic-neuroendocrine tumor (GEP-NET) classification criteria (2010) [20], and GAC was classified by Union for International Cancer Control (UICC) classification criteria (2010, 7th edition) [21]. The GNEC inclusion criteria used the following parameters: (1) a postoperative pathological diagnosis of GNEC after 2010 and (2) a postoperative pathological diagnosis of GNEC with neuroendocrine differentiation before 2010 and then re-diagnosis as GNEC. The GAC inclusive criteria used the following parameters: (1) those whose endoscopic biopsy results and postoperative pathological results were all GAC and (2) those without distant metastasis, as assessed by preoperative examinations. The exclusion criteria used the following parameters: (1) patients receiving neo-adjuvant chemotherapy; (2) intraperitoneal dissemination or distant metastasis observed during surgery; (3) incomplete data for pathological diagnosis; (4) combination with other tumor; or (5) postoperative pathology diagnosed with non-R0 resection. The study was approved by the ethics committee of the Fujian Medical University Union Hospital. Written consent was provided by the patients for their information and specimens to be stored in the hospital database and used for research.

### Variables and definitions

GNEC diagnosis criteria: The diagnosis was mainly based on morphological features of hematoxylin-eosin (HE) staining and specific neuroendocrine markers of immunohistochemistry, including chromogranin A (CGA), synaptophysin (SYN), neuro-specific enolase (NSE) and CD56. Two experienced pathologists evaluated the patient samples (4-μm paraffin sections, HE staining) and confirmed the pathological data of the patients. The projects included immunohistochemical results, mitotic count and tumor cell types. GNEC (WHO G3) was defined as a simple neuroendocrine carcinoma (Ki-67 labeling index > 20% or mitotic count > 20/HPF) or mixed gonadal neuroendocrine carcinoma (Ki-67 labeling index > 20% or mitotic count > 20/HPF) [22]. OS was recorded from the operation time to the last follow-up, date of death or the deadline of the follow-up database (such as lost to follow-up or other causes of death). RFS was the time from the first diagnosis to the initial recurrence. PRS was the time from the initial recurrence to the date of death. The following definitions of recurrent tumors were utilized: (1) local recurrence - gastric stump or anastomotic recurrence, metastasis to lymph nodes around the stomach; (2) hematogenous metastasis - metastasis of remote organs (e.g., the liver, lung, brain, bone); (3) peritoneal recurrence - a tumor derived from the peritoneum or ovary; or (4) metastasis to distant lymph nodes - metastasis to periaortic lymph nodes or lymph nodes outside the abdominal cavity [23]. N0 indicated no lymph node metastasis, and N+ indicated lymph node metastasis.

### Therapeutic approach

Surgical procedure: the patients were subjected to intravenous-inhalation combined anesthesia and signed operation consent documents. The surgical methods, including radical total gastrectomy, radical distal gastrectomy and proximal gastrectomy, were selected depending on the tumor location. Lymph node dissection was performed according to Japanese gastric cancer treatment guidelines (13th edition) [24].

Postoperative adjuvant chemotherapy: patients at stage II or above received chemotherapy based on 5-Fu. The scheme of postoperative adjuvant chemotherapy was Xeloda/S-1 combined oxaliplatin 2-4 weeks after surgery, with a 3-week intermission and a total course of 6 cycles.

Treatment for recurrence: patients without postoperative adjuvant chemotherapy received chemotherapy based on 5-Fu plus platinum. Patients with recurrence after postoperative adjuvant chemotherapy were treated with chemotherapy based on 5-Fu plus Taxol or Xeloda/S-1 plus platinum/Taxol.

### Follow-up

All patients were systematically followed up every 3 months for the first two years and at 6-month intervals thereafter *via* outpatient serviced, visits, letter or telephone. All patients were routinely subject to physical examinations, laboratory tests (e.g., CA19-9, CA72-4, CEA), chest radiography, abdominal CT scan and abdomen ultrasound every year.

### Statistical analysis

Statistical analyses were performed using SPSS 18.0 (SPSS Inc, Chicago, IL, USA). Continuous variables were assessed by $\bar{x}$ s. Categorical variables were calculated using the Pearson *χ2* test or Fisher's test. Survival curves were explored using the Kaplan-Meier method. Comparison of survival rates was performed using the log-rank test. Multivariate analysis of prognostic factors was calculated using Cox proportional hazard models. Statistical significance was set as *p*-values < 0.05.
